# Mixing modes in a population-based interview survey: comparison of a sequential and a concurrent mixed-mode design for public health research

**DOI:** 10.1186/s13690-017-0237-1

**Published:** 2018-01-04

**Authors:** Elvira Mauz, Elena von der Lippe, Jennifer Allen, Ralph Schilling, Stephan Müters, Jens Hoebel, Patrick Schmich, Matthias Wetzstein, Panagiotis Kamtsiuris, Cornelia Lange

**Affiliations:** 1 Department of Epidemiology and Health Monitoring, Robert Koch Institute, Berlin, Germany; 2 Charité – Universitätsmedizin, Berlin, Germany; 3 FG 23 Monitoring Studies and Survey Methods, Robert Koch Institute, P.O. Box 650261, G-13302 Berlin, Germany

**Keywords:** Population health monitoring, Health interview surveys, Mixed-mode designs, Self-administered questionnaires, Sequential mixed-mode design, Concurrent mixed-mode design

## Abstract

**Background:**

Population-based surveys currently face the problem of decreasing response rates. Mixed-mode designs are now being implemented more often to account for this, to improve sample composition and to reduce overall costs. This study examines whether a concurrent or sequential mixed-mode design achieves better results on a number of indicators of survey quality.

**Methods:**

Data were obtained from a population-based health interview survey of adults in Germany that was conducted as a methodological pilot study as part of the German Health Update (GEDA). Participants were randomly allocated to one of two surveys; each of the surveys had a different design. In the concurrent mixed-mode design (*n* = 617) two types of self-administered questionnaires (SAQ-Web and SAQ-Paper) and computer-assisted telephone interviewing were offered simultaneously to the respondents along with the invitation to participate. In the sequential mixed-mode design (*n* = 561), SAQ-Web was initially provided, followed by SAQ-Paper, with an option for a telephone interview being sent out together with the reminders at a later date. Finally, this study compared the response rates, sample composition, health indicators, item non-response, the scope of fieldwork and the costs of both designs.

**Results:**

No systematic differences were identified between the two mixed-mode designs in terms of response rates, the socio-demographic characteristics of the achieved samples, or the prevalence rates of the health indicators under study. The sequential design gained a higher rate of online respondents. Very few telephone interviews were conducted for either design. With regard to data quality, the sequential design (which had more online respondents) showed less item non-response. There were minor differences between the designs in terms of their costs. Postage and printing costs were lower in the concurrent design, but labour costs were lower in the sequential design. No differences in health indicators were found between the two designs. Modelling these results for higher response rates and larger net sample sizes indicated that the sequential design was more cost and time-effective.

**Conclusions:**

This study contributes to the research available on implementing mixed-mode designs as part of public health surveys. Our findings show that SAQ-Paper and SAQ-Web questionnaires can be combined effectively. Sequential mixed-mode designs with higher rates of online respondents may be of greater benefit to studies with larger net sample sizes than concurrent mixed-mode designs.

**Electronic supplementary material:**

The online version of this article (10.1186/s13690-017-0237-1) contains supplementary material, which is available to authorized users.

## Background

Empirical research in public health is frequently based on data derived from population-based health interview surveys (HIS), in which participants are questioned about health issues by an interviewer or fill out self-administered questionnaires. Population-based health surveys can be used to observe prevalence rates for diseases, aspects of medical care and the consequences of diseases as well as the psychological, behavioural and social determinants of health across different population groups. When planning population-based (health) surveys, different aspects of survey design have to be taken into account and this includes sampling procedures and data collection methods. Surveys need to be designed in a manner that minimizes total survey error [1, 2] and that enables them to be undertaken within an acceptable length of time on limited financial budgets. Survey errors can result from various aspects: low sample sizes can lead to inaccurate estimates (sampling error); errors in the sampling procedure may mean that the sample no longer properly reflects the target population (coverage error); a study may be affected by systematic non-response (non-response bias); or systematic errors may have occurred during measurement of the parameters under study (measurement bias). Importantly, the decreasing response rates facing many kinds of survey since the 1990s [3–5] leads to a higher probability of systematic errors such as selection bias, i.e. a certain population group can be underrepresented in an achieved sample due to a particular low response to the survey in this group. In turn, this situation makes it more difficult to gain sufficient sample sizes that are representative of the target population and to do so within the limited financial resources available to the study.

The German Health Update (GEDA) is an example of one such population-based HIS [6]. The study is undertaken as a part of the German Health Monitoring System [7], which is administered by the Robert Koch Institute (RKI) in Berlin. Between 2008 and 2013, three waves of the GEDA study were carried out as computer-assisted telephone interview surveys (CATI) with a random telephone sample that excluded mobile phone numbers. Several problems such as declining response rates, growing selection and coverage bias as well as increasing costs led to the decision to change the study design in a number of ways. On the one hand, a register-based sample was employed as part of a mixed-mode design instead of a random selection of telephone numbers. Moreover, expensive telephone-based interviews were replaced as part of the GEDA study with self-administered paper-based questionnaires (SAQ-Paper) and self-administered online questionnaires (SAQ-Web) [8]. The findings presented here are the result of the research conducted to find the most appropriate way of implementing this mixed-mode design.

Mixed-mode designs are increasingly employed in social and public health research [9, 10]. They offer a combination of survey methods, and this is said to provide a balance between the benefits and drawbacks associated with the choice of a more traditional survey design [5, 11–14]. Research has shown that mixed-mode designs can reduce costs, improve data quality, and can lead to higher response rates as well as a better sample composition [9, 10, 15–22]. However, whether the benefits of a mixed-mode design can be reaped largely depends on the choice of the mixed survey methods, the way in which they are combined and the single modes with which they are compared. Nevertheless, the offer of a choice between survey modes [23–25] can increase the study’s acceptance [11] and the motivation to participate among certain population groups [9, 21, 26]. With regard to total survey error, the possible benefits of a mixed-mode design need to be compared with the potential for errors that can occur due to the particular combination of survey modes. This primarily involves systematic measuring errors that can occur due to the way that mode effects lead questions to be answered differently [5, 9]. The effects of social desirability, which can be caused by the presence of an interviewer, or those related to response scales depending on whether they are presented visually or audibly, are particularly important [e.g. 3, 9, 10, 26]. As a whole, when modes differ at the two levels (e.g. interviewer and oral versus self-administered and visual), the responses varies more than when modes differ only at one level [5].

The offer of both SAQ-Paper and SAQ-Web provides an attractive combination of methods that also takes total survey error into account. These methods generate comparable results [27, 28] because they share a number of characteristics: both are conducted without the presence of an interviewer, and both use visual presentation. This has also been found among adults in Germany, as we have reported in a previous article based on data from a methodological pilot study conducted within the programme of the above-mentioned GEDA study [29]. The findings indicate that mixing self-administered modes, such as SAQ-Paper and SAQ-Web, may be a combination to minimize mode differences in mixed-mode health interview surveys.

SAQ-Web is a very attractive survey mode for large-scale surveys as it results in lower costs and better data quality compared to other methods [9, 10, 12, 27, 30]. However, SAQ-Web can be problematic for representative population-based surveys: it often leads to lower response rates and a lower level of participation among elderly people and people with low levels of education [3, 22, 31–35]. Thus, the use of online questionnaires in population-based studies is only recommended as part of a mixed-mode design [33]; therefore, online questionnaires need to be used together with another mode that is linked to higher response rates and better sample coverage such as SAQ-Paper [12].

Alongside the choice of a combination of methods, the way in which survey modes are presented to respondents is also of paramount importance. In concurrent mixed-mode designs, respondents are offered a choice of different possibilities for participation simultaneously. In a sequential mixed-mode design, the different modes are offered one after the other. Respondents are initially invited to participate through one mode, whereas during one or more additional contacts, non-respondents are invited to participate through additional survey modes. In sequential designs, the least expensive mode is usually offered first in order to reduce overall survey costs [11, 14, 24, 31]. Direct empirical comparisons of sequential and concurrent mixed-mode designs are quite rare. Most methodological studies of mixed-mode survey designs compare survey quality indicators such as response rates between concurrent or sequential mixed-mode designs and a single-mode design. Sequential mixed-mode designs are presumed to be more effective in terms of response rates than concurrent mixed-mode designs; this may be because concurrent designs place an additional burden on respondents to choose their preferred mode of participation [36]. This problem is known as the ‘paradox of choice’ effect [37] and it refers to the overload caused by the pressure to decide between one of many available options. In the survey context, this problem can result in non-participation. In line with the lower response rates expected with online surveys, concurrent [38] and sequential [34] mixed-mode designs implementing SAQ-Paper provide improved response rates compared to single-mode SAQ-Web surveys [31, 34]. However, other studies report a decreased response rate compared to a single-mode SAQ-Paper design in cases where online questionnaires were provided as a concurrent option [15, 23, 36, 39–41] and in a sequential design that began by offering an online questionnaire [42]. One study compared two sequential mixed-mode designs; one offered SAQ-Web followed by SAQ-Paper, the second provided SAQ-Paper before SAQ-Web. SAQ-Web led to the lowest response rates in both survey designs; however, higher online participation was gained in the study that offered SAQ-Web first [31]. Summing up the results of these comparisons, survey quality indicators such as response rates or sample composition differ depending on the mode used in the case of a single mode, and the mix of modes that is adopted (the modes themselves, and, in sequential designs, the order in which the modes are offered).

Only a small number of studies have compared concurrent and sequential mixed-mode designs directly with one another with regard to survey quality indicators such as overall response rates, representativeness and costs, and conclude which one is ‘better’. Millar and Dillman (2011) conducted two experiments investigating how response rates can be improved through the web when mixed-mode designs are employed [24]. They surveyed an internet-savvy population of undergraduate students with internet access, and found that a concurrent design did not outperform a paper-only option. When offering these modes as part of a sequential design, with the web option offered first followed by a postal option, the overall response rate was equivalent to using SAQ-Paper only, but did increase the number of web-based responses.

Another study compared different kinds of concurrent and sequential designs [43]. It included several designs aimed at finding the best way of integrating online questionnaires into the American Community Survey. The results showed that offering a choice between internet and postal questionnaires as part of a concurrent mixed-mode design achieved response rates similar to those of a mail-only strategy with no decrease caused by the online option. This occurred no matter how prominently the choice of options was advertised (in other words, whether the web option was presented on all documents or merely on the paper-and-pencil questionnaire). When offering the two modes as part of a sequential design with the web option offered first, followed by paper questionnaires, only a ‘push accelerated strategy’ with a 2-week interval between the two modes obtained a higher response rate than a mail-only strategy. Adding another week before sending out a reminder together with the other mode achieved the lowest response rate of all [43].

No differences between the two mixed-mode designs were found in a methodological mixed-mode experiment conducted as part of round four of the European Social Survey in the Netherlands. Two randomly divided samples (a sequential mixed-mode design with a web option first, followed by a telephone interview option, followed by a face-to-face interview within a concurrent mixed-mode design) found almost identical response rates with a few more web interviews gained through the sequential design [5].

Many of the published studies tested different modes in various settings and samples, so that the results are difficult to generalize from in terms of population-based health surveys. In our case, a higher rate of online respondents would be important so as to reduce costs. However, implementing online questionnaires risks decreased response rates, especially within a concurrent mixed-mode design. In the light of a lack of direct empirical comparisons between concurrent and sequential mixed-mode designs, we conducted the methodological GEDA 2.0 pilot study described in this paper to provide an indication of which of two particular mixed-mode designs would be the most appropriate for the future GEDA study and employed different survey quality indicators to do so.

## Methods

### Aims and research questions

In addition to testing the practical implementation of a mixed-mode design for the new GEDA wave and testing various methodological concepts and instruments, we conducted a methodological pilot study (GEDA 2.0) that tested the use of concurrent versus sequential mixed-mode designs as part of a health interview survey of adults. Both designs offered two kinds of self-administered questionnaires (SAQ-Web and SAQ-Paper) as well as computer-assisted telephone interviewing (CATI). The aim was to determine whether one of these mixed-mode designs outperformed the other on different survey quality indicators. The study was approved by the German Federal Commissioner for Data Protection and Freedom of Information and informed consent was obtained from all participants. Participants were informed about the goals and contents of the study, about privacy and data protection, and that their participation in the study was voluntary.

The study focused on a comparison of concurrent and sequential mixed-mode survey designs and considered five questions:

- Does a concurrent and sequential mixed-mode design that uses three modes (SAQ-Paper, SAQ-Web and CATI) lead to different response rates?
- What are the socio-demographic characteristics of the achieved samples from the concurrent and sequential mixed-mode designs, and do they differ?
- Are there differences between the two designs with respect to basic health indicators?
- Do the two designs differ in terms of item non-response?
- What are the costs of each study design, and is it possible to identify a more economical mixed-mode design?

### Study design and fieldwork

The study design was chosen so that a sequential mixed-mode design comprising SAQ-Web, SAQ-Paper, and CATI could be compared with a concurrent mixed-mode design that used an identical mode mixture. A telephone interview was offered in order to determine whether it would be an accepted mode as part of this new sampling strategy.

The study was based on a random sample of adults registered with the local resident registries of six municipalities in Germany. The sample covered urban and rural locations as well as eastern and western regions. Participants were selected using a disproportionate stratified random sampling procedure that ensured an equal distribution of men and women, and an equal distribution of age groups (18–29 years, 30–44 years, 45–64 years, and 65–79 years). Local registry offices were asked to provide the name, address, year of birth, and sex of the people in the sample. Telephone numbers were unavailable as these are not recorded on German registers. The gross sample of 6720 participants was randomly allocated to the two mixed-mode survey designs, stratified by region, sex and age. An equal distribution of these factors in each mixed-mode design was important for the analysis and comparison of the two survey designs.

**Fig. 1:**
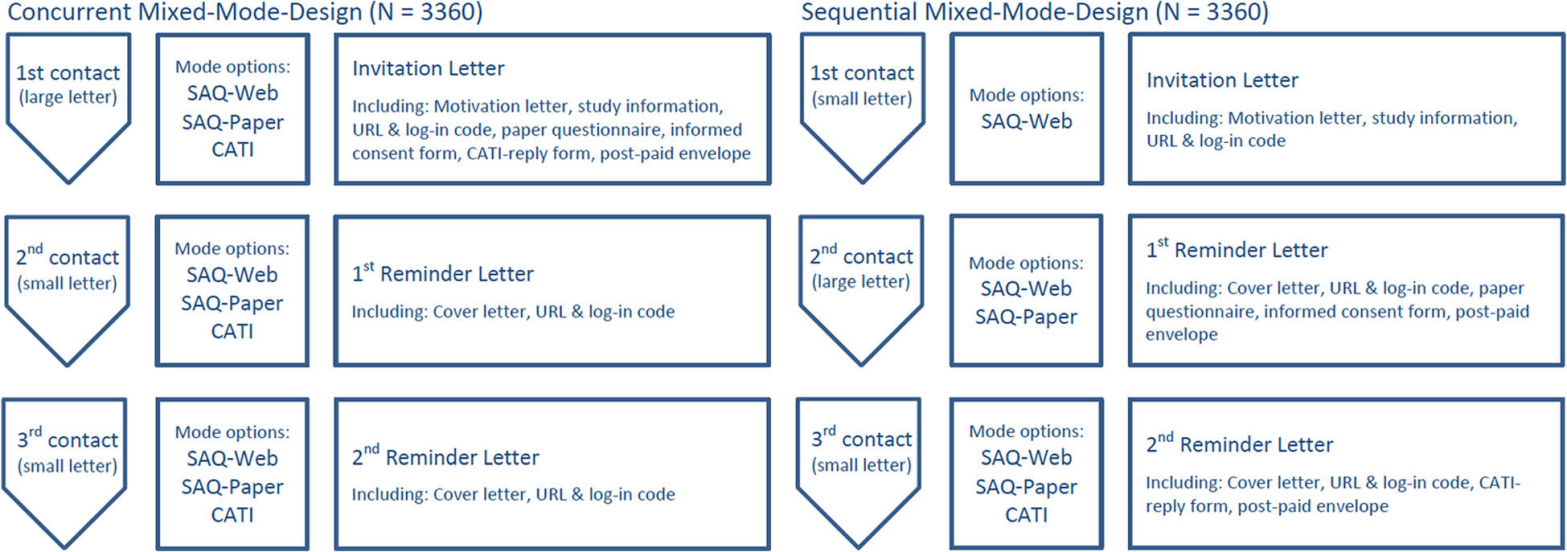
Schematic overview of the GEDA 2.0 methodological pilot study

The procedure for contacting participants in the concurrent mixed-mode design (Fig. 1) consisted of an invitation letter, information about the study and data privacy, the paper questionnaire, a consent form, and a user name and password to access the web option. Respondents were also able to participate through CATI, but had to provide their telephone number (either by phone, email or reply form). Three weeks after the first contact, a reminder was sent out to the non-respondents. User names and passwords were resent, but without a copy of the paper questionnaire. Three weeks later, non-respondents were contacted a third time with a further reminder. In the concurrent mixed-mode design, the reminder included the cover letter and the respondent’s user name; in the sequential mixed-mode design, a CATI reply form and a pre-paid envelope was also sent out as part of the second reminder.

Participants assigned to the sequential mixed-mode design received information about the study and a user name and password in their first contact letter (Fig. 1). Non-respondents received a paper version of the questionnaire 3 weeks later and were invited to participate by completing the paper or web questionnaire. After three further weeks, a third letter was sent to non-respondents suggesting a telephone interview. In this case, respondents completed a form providing their telephone number and returned it to the Robert Koch Institute.

There is no register of telephone numbers in Germany that includes both publicly listed and unlisted numbers that could have been used to match the register-based sample. Moreover, far fewer telephone numbers are now listed in the directories in Germany than during the 1990s and this change has been accompanied by a dramatic increase in ‘non-pubs’ (non-published phone subscribers). Serious problems would have arisen if these telephone directories had been used. Unlisted subscribers differ in certain socio-demographic characteristics: they are more likely to live in urban than rural areas, are younger and have a higher level of education [44]. Moreover, matching some sections of the sample with the public telephone directory would not have enabled any general conclusions to be drawn from the findings, since the sample would have been extremely biased. In addition, although it would have been possible to match addresses with commercial registers of telephone numbers, doing so would have been costly and fairly ineffective (it is associated with a 20% success rate).

The questionnaires used in the different modes were designed using a combination of unified-mode-design (to ensure that the questionnaires were almost identical) – this reduced mode-effects – and mode-specific-design so as to capitalize on the advantages provided by modes that lead to better data quality [17]. In all modes, standardized formulations and explanations were used. Telephone and online questionnaires were optimized using filter skips whenever this did not impair the understanding of a filter question. Plausibility checks and do-answer-checks were also integrated into the online questionnaires. The average response time for the online questionnaire was 26 min. The dropout rate among people who started the web questionnaire was just 2%. On average, the telephone interviews lasted 35 min.

### Response rates

The response rates for each mixed-mode design were calculated according to the standards of the American Association for Public Opinion Research (AAPOR). The response rate was calculated as the share of interviews from the gross sample (AAPOR-Response Rate 1) [45]. Completed questionnaires were only considered valid if the respondents had signed an informed consent form before participating in the study. This consent form had to be returned in a separate envelope. During the web questionnaire, participants were asked to tick a box indicating whether they had read and accepted the study’s data protection conditions. The overall response rate for both designs was 18.3%.

### Sample composition

We performed a two-level comparison of the two samples’ various socio-demographic characteristics. The first step involved comparing the achieved net samples of each mixed-mode design with the original gross sample according to sex, age, region of residence, and the type of residential area. These characteristics were chosen as the distribution of these characteristics in the gross sample was also known. The second step involved analysing the statistical differences between the two designs; we included current employment status, marital status, type of household, migration background, educational level [46] and income level (based on household net income and using income quintiles).

### Health indicators and data quality

We analysed a set of health-related indicators for differences in crude prevalence rates and rates of item non-response. The prevalence rates for 12-month diagnoses of diabetes, hypertension, coronary heart disease, and/or chronic bronchitis were calculated using a question about whether participants had ever been diagnosed by a doctor with one of these diseases. Respondents who answered ‘yes’ were asked whether they had suffered from this disease during the past 12 months. The prevalence of obesity was assessed by calculating body mass index (BMI) using the World Health Organization’s recommendations (BMI ≥30 kg/m2) [47] based on self-reported weight and height. Self-rated health was assessed using the first question asked as part of the Minimum European Health Module [48]. Current depression was evaluated using the diagnostic algorithm set out in the eight-item Patient Health Questionnaire depression scale (PHQ-8) [49]. Current mental wellbeing was assessed using the five-item WHO Well-Being Index (WHO-5) [50]. Finally, perceived social support was measured using the three-item Oslo Social Support scale (OSS-3) [51]. In addition, data was collected on the following forms of health-related behaviour: current smoking status, at-risk alcohol consumption (measured by AUDIT-C) [52], sporting activity in the last 3 months, and vaccination status.

### The scope of fieldwork and cost analyses

We calculated the overall costs of the two mixed-mode designs based on postage, printing and data entry costs and time requirements. We also performed a modelling calculation that reproduced the real field conditions associated with a population-based survey aimed at gaining a 20,000 net sample with a supposed response rate of 20% for a sequential mixed-mode design; the aim here was to obtain a better assessment of the cost-efficiency of the two designs. The cost analysis and the scope of fieldwork are described in the results.

### Statistical analysis

To test for statistically significant differences, we calculated response rates for different sample characteristics, crude (unadjusted) prevalence rates for health indicators, and prevalence rates of item non-response by survey-design. Pearson’s χ^2^-tests were used to test for statistically significant differences (α = 0.05). No adjustments were made to significance levels, which could have helped control for multiple testing, as this ensured that the analysis remained sensitive to possible differences between the two designs. Statistical analyses were conducted using STATA 14.1 SE (StataCorp LP, College Station, TX).

## Results

### Response rates

Table 1 shows the disposition codes (a code for the result of the contact) and response rates for the two mixed-mode designs. From the total 1320 questionnaires that were returned (594 in the sequential design and 726 in the concurrent design), 61 were discarded because a respondent’s date of birth was inconsistent with that provided by the registry office. In addition, 81 interviews were not analysed as they were not accompanied by a signed informed consent form; all of these were paper questionnaires. In total, 1178 valid questionnaires were analysed: 561 were obtained from the sequential mixed-mode design and 617 stemmed from the concurrent mixed-mode design.

**Table 1:** Disposition codes and response rates by survey design

		Concurrent mixed-mode design			Sequential mixed-mode design		
	AAPOR-code	n	% gross sample	% clean gross sample	n	% gross sample	% clean gross sample
Gross sample		3360	100%		3360	100%	
Not Eligible							
No eligible respondent	4.70	140	4.2%		154	4.6%	
Clean gross sample		3220	95.8%	100%	3206	95.4%	100%
Complete interviews	1.0	617		19.2%	561		17.5%
Eligible, “Non-Interview”							
Refusal	2.11	183		5.7%	177		5.5%
Break-off or partial with insufficient information	2.12	8		0.2%	16		0.5%
Non-Contact: Nothing ever returned	2.21	2272		70.6%	2387		74.5%
Respondent unavailable during field period	2.25	8		0.2%	5		0.2%
Physically or mentally unable	2.32	23		0.7%	27		0.8%
Miscellaneous	2.36						
Signed privacy declaration not returned	–	51		1.6%	30		0.9%
Questionnaire returned with wrong birthdate (wrong Respondent)	–	58		1.8%	3		0.1%
Response rate^a^				19.2%			17.5%

^a^“Response rate 1” in accordance with the American Association for Public Opinion Research (2011). *p* = 0.085 (chi-square)

The response rate was 19.2% in the concurrent mixed-mode design compared with 17.5% in the sequential design. There was no statistically significant difference between these response rates (*p* = 0.085).

We also calculated response rates by age group (Table 2). The differences between the two designs were small and statistically insignificant.

**Table 2:** Response rates by survey design and age group

	Concurrent mixed-mode design (*n* = 617) %	Sequential mixed-mode design (*n* = 561) %	*p*-value
18–29 years	12.2	13.2	n.s.
30–44 years	17.9	14.9	n.s.
45–64 years	23.4	21.9	n.s.
65–79 years	22.6	19.8	n.s.
Total	19.2	17.5	n.s.

*n.s*. not significant (*p* > 0.05)

### Sample composition

Table 3 presents the socio-demographic characteristics of the gross and net samples according to the type of mixed-mode design. The results show that women, older people, and people living in urban areas were significantly overrepresented in the net samples gained for both designs compared to the original gross sample. People living in eastern Germany were significantly overrepresented in the net sample of the sequential mixed-mode design. However, when the two net samples were compared, no significant differences were found with regard to sex, age, region of residence and type of residential area (*p*-values > 0.05).

**Table 3:** Socio-demographic characteristics of the achieved net samples, compared with the original gross samples

	Concurrent mixed-mode design			Sequential mixed-mode design		
	Gross sample (*N* = 3220)	Net sample (*n* = 617)	*p*-value	Gross sample (*N* = 3206)	Net sample (*n* = 561)	*p*-value
Sex (%)						
Men	50.1	45.1	<0.05	49.7	42.6	<0.05
Women	49.9	54.9		50.3	57.4	
Age group (%)						
18–29 years	23.9	15.2	<0.05	24.3	18.4	<0.05
30–44 years	25.2	23.5		24.8	21.0	
45–64 years	25.5	31.1		25.5	31.9	
≥ 65 years	25.5	30.1		25.4	28.7	
Region of residence (%)						
East	49.3	53.3	n.s.	49.1	55.4	<0.05
West	50.7	46.7		50.9	44.6	
Residential area (%)						
Urban	51.1	59.0	<0.05	50.6	58.3	<0.05
Rural	48.9	41.0		49.4	41.7	

*p*-value: test of significance between clean gross sample and net sample. *n.s*. not significant (*p* > 0.05)

Further comparisons could be made only for the achieved samples of the two mixed-mode designs as the required (self-reported) data was only collected using the questionnaires employed in the two surveys. The achieved samples for the mixed-mode designs showed no significant differences when compared by age, marital status, household type, educational level, income, employment status or family history of migration (Table 4).

**Table 4:** Socio-demographic characteristics of the achieved samples^a^

	Concurrent mixed-mode design (*n* = 617)	Sequential mixed-mode design (*n* = 561)	*p*-value
Age (years)			
Range	18–79	18–79	
Median	51.0	51.0	
Mean (SD)	51.0 (17.0)	50.1 (17.8)	n.s.
Marital status (%)			
Married and cohabitating	61.2	59.1	
Separated/divorced/widowed	15.3	13.8	
Single	23.5	27.1	n.s.
Household type (%)			
One-person household	20.3	18.7	n.s.
Educational level (%)^b^			
Low	20.4	20.6	
Medium	51.6	51.4	
High	28.0	28.0	n.s.
Equivalent income (€)			
Range	92–6750	40–7333	
Median	1333	1333	
Mean (SD)	1527 (900)	1512 (891)	n.s.
Equivalent income (%)^c^			
< 60% of median income	24.1	26.3	
60–150% of median	62.9	61.3	
≥ 150% of median	12.9	12.3	n.s.
Employment status (%)			
Working	58.0	60.0	n.s.
Migration background (%)			
Yes	10.3	12.2	n.s.

*n.s*. not significant (*p* > 0.05); *SD* standard deviatio

^a^No information was available for these parameters for the original gross sample

^b^Education level: CASMIN (Comparative Analysis of Social Mobility in Industrial Nations)

^c^Equivalent income based on the Organization for Economic Cooperation and Development scale; median according to EU-SILC 2010

### Health indicators and data quality

**Fig. 2:**
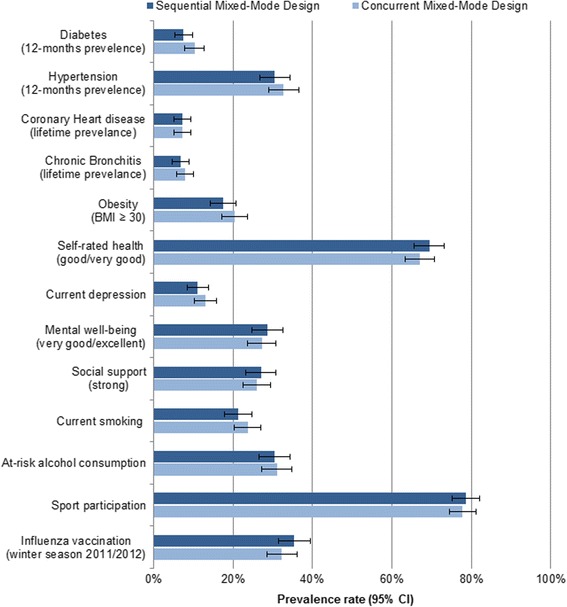
Crude prevalence rates (95% confidence intervals) for basic health indicators by mixed-mode design

Given that mixed-mode designs are used in public health surveys, we aimed to determine whether concurrent and sequential mixed-mode designs lead to significantly different results. The crude (unadjusted) prevalence rates for a number of essential health indicators are presented separately for each design in Fig. 2. A comparison of the mixed-mode designs found no statistically significant differences between any of the health indicators (*p*-values > 0.05).

Comparison of the missing values for some essential indicators is used as a measure of data quality (Table 5). For the 12-month prevalence of diabetes, the percentage of missing values was significantly higher in the con current design than in the sequential design. Similar differences were observed for the 12-month prevalence of hypertension, the lifetime prevalence of chronic bronchitis and the sports participation rate. No statistical differences in missing values for any other indicators were found between the two designs. The higher levels of missing values in the concurrent design were due to the higher proportion of paper questionnaires [see Additional file 1: Table S1].

**Table 5:** Missing values for health indicators by survey design

	Concurrent mixed-mode design	Sequential mixed-mode design	
	n (%)	n (%)	*p*-value
Diabetes (12-month prevalence)	36 (5.8)	18 (3.2)	0.031
Hypertension (12-month prevalence)	40 (6.5)	18 (3.2)	0.009
Coronary heart disease (lifetime prevalence)	7 (1.1)	4 (0.7)	n.s.
Chronic bronchitis (lifetime prevalence)	37 (6.0)	17 (3.0)	0.015
Obesity (BMI ≥30)	10 (1.6)	18 (3.2)	n.s.
Current depression	47 (7.6)	39 (7.0)	n.s.
Mental well-being	30 (4.9)	32 (5.7)	n.s.
Social support	9 (1.5)	10 (1.8)	n.s.
Current smoking	13 (2.1)	11 (2.0)	n.s.
At-risk alcohol consumption	53 (8.6)	39 (7.0)	n.s.
Sport participation	13 (2.1)	3 (0.5)	0.020
Influenza vaccination (2011/2012)	20 (3.2)	10 (1.8)	n.s.

*n.s* not significant (*p* > 0.05)

### Participation by mode and cost analysis

**Fig. 3:**
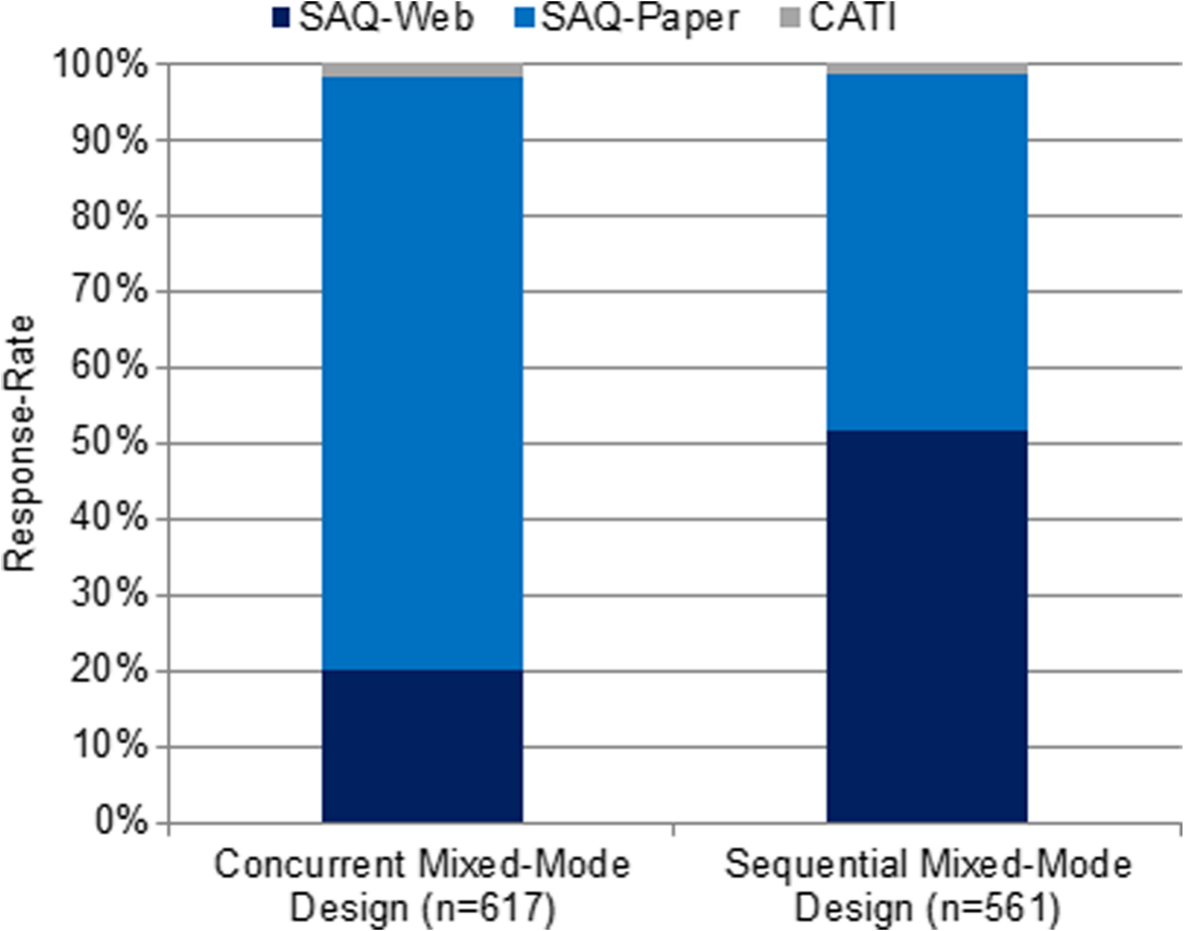
Participation in the mixed-mode designs by survey mode. SAQ-Paper = self-administered paper questionnaire; SAQ-Web = self-administered web questionnaire; CATI = computer-assisted telephone interview

**Fig. 4:**
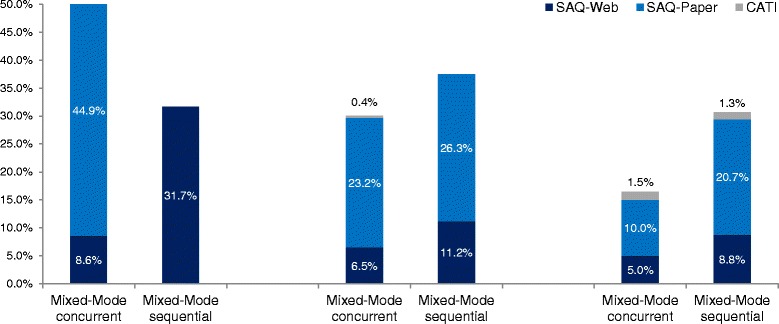
Timeline of valid questionnaires obtained by mixed-mode design and number of contacts. SAQ-Paper = self-administered paper questionnaire; SAQ-Web = self-administered web questionnaire; CATI = computer-assisted telephone interview; percentages according to mixed-mode-designs

Before reporting a detailed description of the costs of each design, we first present the distribution of the survey modes within the mixed-mode designs (Fig. 3) and the number of contacts needed to gain the specified number of returned questionnaires (Fig. 4). In the concurrent mixed-mode design, about 20% of valid responses (*n* = 124) were gained via SAQ-Web, compared with more than 50% of valid responses (*n* = 290) in the sequential design. In both designs, the CATI option was chosen by less than 2% of the participants who provided a valid response (concurrent: *n* = 11, sequential: *n* = 7) (Fig. 3). Therefore, offering a telephone interview as part of this sampling strategy proved to be ineffective.

There was a pronounced difference between the contacting waves for the two mixed-mode designs with regard to the way in which the respondents participated (Fig. 4). This difference is likely to have been caused by the difference in modes that were offered: the highest response rate occurred when SAQ-Paper was offered to the respondents. More than half of the returned concurrent design questionnaires (more than 85% of paper questionnaires) were received from the first wave of invitations. In the sequential design, the first contact only produced about one-third of the questionnaires (online questionnaires). The majority of questionnaires in the sequential design were returned after the second contact (after the paper version had been sent to the respondents). A high proportion of respondents returned paper questionnaires. In the concurrent design, where the second contact only included a reminder and the user name, another 30% answered; once again, a large proportion provided a paper questionnaire. The third contact letter was identical in both designs: it stated the URL and the user name for the online questionnaire. About 17% of the questionnaires in the concurrent and 31% in the sequential mixed-mode designs were obtained during the last few weeks of the study. After the third contact had been made, about two-thirds of respondents had already returned their paper questionnaires for the concurrent and sequential designs.

The costs of the web questionnaire were comparably low, mainly because manual data entry was unnecessary. All data entered online were automatically saved in a database, meaning that the high costs incurred by data entry were avoided. Excluding the infrastructure and overhead costs (e.g., server space, programming work etc.), which are similar to those incurred during the preparation of a paper questionnaire, the only time consuming function undertaken for the fieldwork was data management. Data management involved managing the server and the questionnaires held in the database.

The cost of the paper questionnaires was significantly higher. Printing costs are a major expense that is simply not incurred by online questionnaires. In this survey, production costs amounted to about EUR 3 per (60-page) questionnaire. The second major expense was postage. The size and weight of the paper questionnaire (including the invitation letter) meant that mailing the concurrent option in all modes (see Fig. 1) was about three times more expensive than mailing the invitation letter containing information on how to take part via the web (URL, username and password). Mailing costs (including filling the envelopes) amounted to EUR 0.70 for a small envelope (invitation letter, online information) and EUR 2.20 for a large envelope (invitation letter and paper questionnaire). Further postal costs were incurred for each returned paper questionnaire. Moreover, as a means of encouraging respondents to return their questionnaires, each mailing was supplied with a pre-addressed postage-paid envelope; this incurred EUR 2.20 in costs per returned envelope.

Another costly and time-consuming step involved manual data entry and the logistics of receiving and sorting questionnaires. The paper questionnaires used in this survey were designed and produced as machine-readable forms. As such, completed questionnaires that were returned to the Robert Koch Institute were scanned, verified and exported to a database. Reviewing and data correction was undertaken manually and could not be entirely automated. The whole process took, on average, 8 minutes per questionnaire plus 1 minute for logistics. Other costs to consider were supervision of the data entry process and management of data quality (e.g., at least 10% of the questionnaires were entered twice to ensure data quality and develop data correction guidelines). Overall, the use of web questionnaires required significantly less labour than paper questionnaires.

The total number of letters sent out was higher in the sequential mixed-mode design, but the number of paper questionnaires was comparatively low given the higher rate of cheaper and smaller postal letters (Table 6). The postage and printing costs incurred by the sequential design were slightly lower than for the concurrent design. As the sequential mixed-mode design received considerably more web questionnaires, the time available for scanning and verifying paper questionnaires was reduced by about 40%. These advantages decreased in light of the 1.7% lower overall response rate gained by the sequential mixed-mode design (Table 6). The overall response rate is also relevant in calculating the costs per valid questionnaire. The postage and printing costs for each valid questionnaire were almost the same for both the sequential design and the concurrent design, but there was still potential to save labour costs associated with about 40% of the questionnaires given the higher proportion of online responses.

**Table 6:** Costs for the concurrent and the sequential mixed-mode designs in GEDA 2.0^a^

	Concurrent mixed-mode design					Sequential mixed-mode design				
	Number of mailings	Cost factors				Number of mailings	Cost factors			
		Small letter	Large letter	Printed questionnaire	Data entry		Small letter	Large letter	Printed questionnaire	Data entry
1st contact	3360		3360	3360		3360	3360			
2nd contact	2786	2786				2991		2991	2991	
3rd contact	2487	2487				2677	2677			
Total letters	8633	5273	3360	3360		9028	6037	2991	2991	
Valid questionnaires SAQ-Paper	482				482	264				264
Valid questionnaires SAQ-Web	124					290				
Valid questionnaires (without CATI)	606					554				
Cost analysis										
Costs per unit		0.70 €	2.20 €	3.00 €	9 min		0.70 €	2.20 €	3.00 €	9 min
Postage and printing costs in €		3691.10	7392.00	10 080.00			4225.90	6580.20	8973.00	
Postage for received question. in €			1060.40					580.80		
Total postage and printing costs in €				22 223.50				20 359.90		
Total costs in working minutes					4338					2376
Total postage and printing costs per valid questionnaire in €				36.67					36.75	
Total working time needed per valid questionnaire (in min)					7.2					4.3

^a^Excluding overhead costs and working time for supervision, data cleaning, and homogenization. *CATI* computer-assisted telephone interviewing

In order to estimate the costs for larger population-based interview surveys, we modelled the calculations using a net sample of 20,000 with a supposed response rate of 20% for the sequential mixed-mode design (Table 7). We assumed a higher response rate (20%) than the methodological study’s real field conditions because of higher than expected respondent motivation. All other components were taken from the findings produced by GEDA 2.0 (Table 6). Both designs incurred very similar levels of postage and printing costs. In a study of this size, more labour is needed in concurrent designs. Given the management of incoming paper-and-pencil questionnaires and data entry demands, the concurrent design required about 960 more working hours than the sequential design. This results in extra costs that have to be paid as well as a time lag in full access to survey data. We calculated labour expenses for data entry based on the pay scale for German public services (Table 7) and found lower labour costs of about EUR 24,000 for the sequential design compared with the concurrent design. In total, the difference in costs between the two designs was less than EUR 25,000, with overall costs of EUR 670,000 for the concurrent and EUR 645,000 for the sequential design (excluding overheads).

**Table 7:** Model calculation for a cost analysis in concurrent and sequential mixed-mode designs (number of cases: 20,000; sequential mixed-mode design response rate: 20.0%)^a^

	Concurrent mixed-mode design					Sequential mixed-mode design				
	Numberof mailings	Cost factors				Numberof mailings	Cost factors			
		Small letter	Large letter	Printed questionnaire	Data entry		Small letter	Large letter	Printed questionnaire	Data entry
1st contact	91 419		91 419	91 419		100 000	100 000			
2nd contact	75 802	75 802				89 018		89 018	89 018	
3rd contact	67 666	67 666				79 673	79 673			
Total letters	234 887	143 468	91 419	91 419		268 690	179 673	89 018	89 018	
Valid questionnaires SAQ-Paper	15 908				15 908	9531				9531
Valid questionnaires SAQ-Web	4092				0	10 469				0
Valid questionnaires	20 000					20 000				
Cost analysis										
Costs per unit		0.70 €	2.20 €	3.00 €	9 min		0.70 €	2.20 €	3.00 €	9 min
Postage and printing costs in €		100 427.74	201 122.11	274 257.43			125 770.83	195 839.29	267 053.57	
Postage for received questionnaires in €			34 996.70					20 967.51		
Total postage and printing costs in €				610 803.97					609 631.20	
Total costs in working minutes					143 168					85 776
Total postage and printing costs per valid questionnaire in €				30.54					30.48	
Total working time needed per valid questionnaire (min.)					7.2					4.3
Total costs for 20 000 valid questionnaires in working hours (working weeks), rounded up					2400 (62)					1450 (37)
Costs for working time in € (per pay scale of public service)^b^					59 182.82					35 318.18
Total postage and printing costs for 20 000 valid questionnaires (in €)				610 803.97					609 631.20	
Total costs for 20 000 valid questionnaires (in €)					669 985.79					644 949.38

^a^Excluding overhead costs and working time for supervision, data cleaning, and homogenization

^b^Calculated with German TVöD 4 (payment 42 000 €/year including social insurance), 44 weeks crude data entry per year

**Fig. 5:**
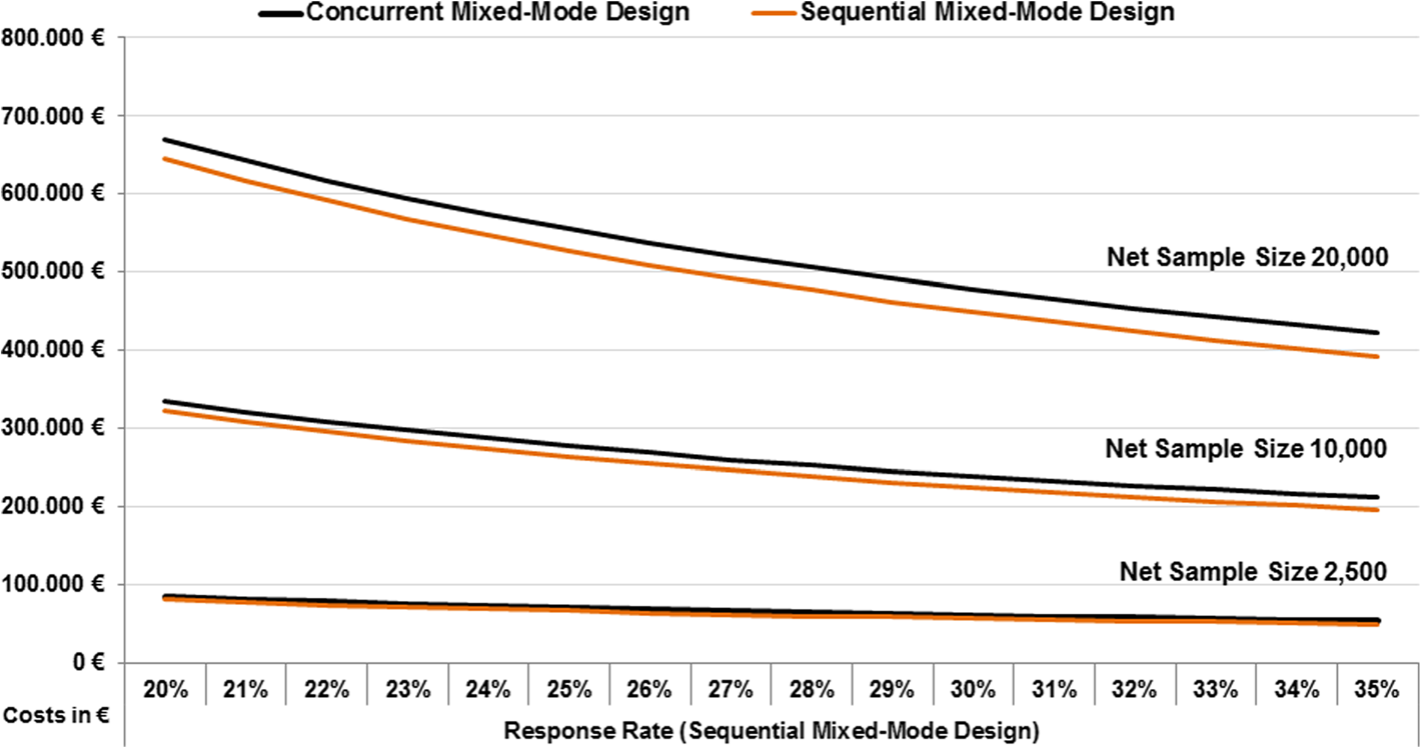
Estimates of costs for concurrent and sequential mixed-mode designs with different response rates and net sample sizes

This model of possible survey costs (which excludes data cleaning and overheads) was used for different net sample sizes and different response rates. For example, we calculated the costs associated with net sample sizes of 20,000, 10,000, and 2500 respondents and for different assumed response rates (ranging from between 20% and 35%). In all three net samples, the difference between the two mixed-mode designs increased with higher response rates, with the sequential mixed-mode design having a slight advantage due to its somewhat lower costs compared to the concurrent mixed-mode design (Fig. 5). In general, the cost differences between the two mixed-mode designs declined with smaller net samples.

## Discussion

This study compared two different mixed-mode designs: a concurrent and a sequential design. The response rates for the two designs were similar with regard to overall response rate and for different population groups. Online respondents represented 50% of participants in the sequential mixed mode design, and 20% of participants in the concurrent mixed-mode design. The concurrent mixed-mode design and the second contact letter in the sequential mixed-mode design offered a paper-and-pencil questionnaire, an option that the participants preferred. An important factor in this preference was the way in which we contacted potential participants. No national list of email addresses was available, and German registry offices do not collect this information; therefore, we contacted potential respondents via mail and invited them to complete an online questionnaire. The invitation that was sent out to potential respondents included a URL that had to be manually typed into a browser. Compared with an email invitation that includes an embedded, hot-linked URL, responding required more effort. Researching email addresses or collecting them in a panel or longitudinal study could be an effective way of increasing online participation while also lowering costs [34, 53].

Millar and Dillman (2011) found that web response rates may increase if sequential designs offer the web option first; this was also confirmed by the results of GEDA 2.0. Millar and Dillman’s experiments were conducted with a highly internet-literate population that had full access to the internet. Offering this mode as part of a concurrent design led to a lower response rate, whereas offering online participation followed by a paper questionnaire in sequential designs achieved similar response rates compared to the responses gained from a mail-only mode [24]. Millar and Dillman also suggest that sequential designs gain the highest level of online participation when reminder emails include a link to an online questionnaire in addition to postal contacts. Revilla (2010) found no differences between concurrent and sequential approaches that offered different modes as part of round four of the European Social Survey in the Netherlands [5]. In a study investigating an internet option provided as part of the American Community Survey [43], the sequential mixed-mode design with an online option achieved a higher response rate than the concurrent design when the time lag between the two contacts was short (2 weeks). The lowest response rates were observed in a sequential design with a time lag of 3 weeks. Technical reasons led us to opt for a three-week interval between the first invitation letter and a reminder offering another mode of participation. Therefore, choosing a shorter period between invitations and reminders, as well as researching email addresses in advance may improve response rates in sequential designs. If concurrent designs are considered under the objective of gaining higher response rates (this is independent of participation mode and ignores survey costs), it may be worthwhile sending out paper questionnaires with all reminders.

In both designs, participation via CATI was extremely low; this was presumably caused by the survey’s procedure (respondents had to return their telephone number by post). This suggests that there is little reason to offer a telephone interview when using a sample from registry offices. This is especially the case when telephone numbers are unavailable beforehand, and respondents have to be contacted by post and asked to provide a telephone number instead of being called directly.

There were no systematic differences between the characteristics of the achieved samples for either of the mixed-mode designs or the prevalence rates of different health indicators. In the literature, SAQ-Paper and SAQ-Web are considered to be equivalent [15]. Nevertheless, the fact that a higher percentage of people participated via SAQ-Web in the sequential design did not lead to significantly different samples. However, these results should be interpreted with caution given the low number of participants. The differences between data collection modes have been published elsewhere [29].

The two designs led to slight and unexpected differences in costs. We assumed that the sequential mixed-mode design would produce lower costs in all cases. However, printing and postage costs were almost the same, whereas labour costs for data entry were about 40% lower. A reason for this was the low response rates generated by the first contact in the sequential design and by the study as a whole. The low response rate after the first contact meant that a large number of more expensive envelopes containing the paper questionnaire had to be sent out for the sequential design. Another important aspect was the decision not to send follow-up paper questionnaires for the concurrent design. If we had also sent out the paper questionnaires with the first reminder, the postage and printing costs would have been much higher for the concurrent design. As expected, labour costs were lower for the sequential design because of the higher proportion of online questionnaires which meant less time had to be spent on data entry.

Overall, our results suggest that concurrent and sequential mixed-mode designs are appropriate for studies of this size and that choosing either of these designs will have little effect on the outcome of a study. However, the higher general overhead costs associated with mixed-mode surveys [5] means that a single-mode SAQ-Paper design may be a better alternative for smaller surveys. Modelling these results against the real field conditions faced by future GEDA surveys (German health interview surveys with 20,000 respondents and an assumed response rate of 20%), led to estimates of slightly higher postage and printing costs when using the concurrent design, albeit with very little actual difference between the two designs. However, a concurrent design may require a longer survey period due to the extra time it involves; time that has to be paid for. As such, the strongest argument for choosing the sequential design for survey fieldwork is that it is faster and, therefore, more cost-effective in this regard than the concurrent design.

The sequential design was also more successful in convincing people to participate online. This played a significant role in gaining higher quality data more quickly, with less logistical effort, and less labour time spent on data entry and validation. Taken together, these factors reduce costs and time. Consistent with most other empirical results, the data quality of the online questionnaires was better than the data gained from the paper questionnaires. In addition, the online questionnaires also showed a significantly lower rate of item non-response. Online questionnaires offer several ways to ensure high data quality. Automated filters and complex routines can minimize the number of unanswered or incorrectly answered items. Plausibility checks are another means of improving data quality. If questions go unanswered, respondents can be interrupted by a popup (do-answer-check) asking whether the previous question was intentionally unanswered or accidently skipped. Plausibility checks can also be used to enforce limits on numeric questions. Such guided answers prevent errors and lead to more accurate data that requires less time and effort to be spent on data cleansing. In the GEDA 2.0 study, these technical measures were only used to a limited extent so as to exclude possible mode differences [29]. The data quality gained by the online questionnaires was still clearly better than that of the paper questionnaires. The American Community Survey also found better data quality when data was acquired online than through the use of paper questionnaires [39]. In addition, less time was needed for data preparation and quality assurance in the case of online questionnaires. Moreover, as there was no need for manual data entry, the likelihood of human error was drastically reduced. Data accuracy is an important benefit associated with web-based questionnaires; however, the costs of data cleaning were not included in the model calculation. These costs are hard to estimate, because data cleaning was undertaken using pooled data gained from both modes.

Based on these results and the experiences gained from other methodological studies, the implementation of an informed sequential design (when an opportunity to opt for another mode is provided on first contact) may be the most appropriate choice for future GEDA waves. Tancreto et al. (2012) experimented with different strategies as part of a concurrent design where they differentiated between prominent and non-prominent choices for the online option. They obtained slightly higher response rates when the choice of participation mode was displayed prominently [43]. Therefore, the first invitation letter should include a user name and password for participating via a web questionnaire, but should also state that in 2 weeks, the respondent will receive a paper version of the questionnaire if he or she is unable or unwilling to use the internet. This approach could dramatically reduce costs and the duration of survey fieldwork in particular compared to a concurrent design. Moreover, response rates may also be higher than in the sequential mixed-mode design put in place in GEDA 2.0.

### Strengths and limitations

The strengths of this methodological pilot study include its randomized study design, the consideration of different regions (eastern and western Germany, urban and rural regions) in the sampling plan, and the large gross sample. Despite these strengths, the results face a number of limitations. One limitation is the relatively small net sample size. Each survey design had a relatively small number of participants, meaning that results based on the net samples should be interpreted with caution. Possible differences between the two mixed-mode designs might have been overlooked owing to a lack of statistical power. The CATI component also faces a further limitation as phone numbers could not be obtained before potential respondents were contacted. In addition, although resending paper questionnaires with the reminders might have improved response rates, it would have also increased costs. Including a single-mode SAQ-Paper control group would have been a helpful way of comparing the two mixed-mode samples with a sample achieved in a single-mode design.

As sampling was based on data gained from registry offices and because the sample was limited to six sample points, it does not make sense to compare the response rates in this study to those of previous GEDA surveys. Previous studies employed population-based samples of telephone numbers drawn from directories alongside randomly generated telephone numbers. Telephone sampling is incompatible with samples based on addresses held by German registry offices.

Other limitations concern the external validity of the results. The pilot study was conducted in a German setting, using registry-based samples of the adult population, and the results cannot simply be transferred to other countries, settings or target populations. In particular, this applies to the cost analysis, which was based on German postage and personnel costs. Moreover, the relatively low overall response rate of 18.3% should be taken into account when considering the generalizability of our results. Systematic non-response may have led to selection bias, which could have affected our results. However, as the literature suggests no clear relation between response rate and representativeness [54, 55], a low response rate does not necessarily lead to selection bias or to a lack of generalizability.

## Conclusions

The results of this study contribute to the research on implementing mixed-mode designs in population-based public health surveys. We found that it was possible to effectively and efficiently combine SAQ-Paper and SAQ-Web questionnaires, with comparable results between concurrent and sequential mixed-mode designs. The two designs were equivalent with regard to response rates and sample composition, but the sequential design showed advantages in item non-response and lower labour costs because of the higher share of web responses. These advantages increased with larger net samples and response rates that favoured the sequential design. For smaller sample sizes, single-mode surveys with paper questionnaires may be more appropriate due to the lower overhead costs. Finally, when large sample sizes are needed for population-based health interview surveys of adults in Germany, the sequential mixed-mode design tested for this study may offer a practical alternative to reducing costs and increasing timeliness, given the higher percentage of online participants (Additional file 2).

## Additional files

Additional file 1: Table S1. Missing values for health indicators by survey design and mode of data collection. (PDF 26 kb)

Additional file 2: Figure A1. Model-adjusted prevalence rates (95% confidence intervals) for basic health indicators by mixedmode design, adjusted for age, sex, marital status, household type, education, income, employment status, and migration background. (TIFF 208 kb)

## Abbreviations

AAPOR: American Association for Public Opinion Research
AUDIT-C: Alcohol Use Identification Test
BMI: Body Mass Index
CATI: Computer-assisted Telephone Interview
GEDA: German Health Update Study
OSS-3: Oslo Social Support Scale
PHQ-8: Patient Health Questionnaire Depression Scale
SAQ-Paper: Self-administered Paper-and-Pencil Questionnaires
SAQ-Web: Self-administered Online Questionnaires
WHO-5: World Health Organization Well-Being Index
