# Supplementary material for: Mixing modes in a population-based interview survey: comparison of a sequential and a concurrent mixed-mode design for public health research
Source: Arch Public Health. 2018 Jan 4;76:8. doi: 10.1186/s13690-017-0237-1 (PMC5791202; doi:10.1186/s13690-017-0237-1)
Supplement: Supplementary file 1 — Missing values for health indicators by survey design and mode of data collection. (PDF 26 kb) [file 13690_2017_237_MOESM1_ESM.pdf]

Table A1. Missing values for health indicators by survey design and mode of data collection

	Concurrent mixed-mode design			Sequential mixed-mode design		
	SAQ-Web (n=124)	SAQ-Paper (n=482)	CATI (n=11)	SAQ-Web (n=290)	SAQ-Paper (n=264)	CATI (n=7)
	n (%)	n (%)	n (%)	n (%)	n (%)	n (%)
**Diabetes (12-month prevalence)**	0 (0.0)	36 (7.5)	0 (0.0)	0 (0.0)	18 (6.8)	0 (0.0)
**Hypertension (12-month prevalence)**	0 (0.0)	40 (8.3)	0 (0.0)	0 (0.0)	18 (6.8)	0 (0.0)
**Coronary heart disease (lifetime prevalence)**	0 (0.0)	7 (1.5)	0 (0.0)	0 (0.0)	4 (1.5)	0 (0.0)
**Chronic bronchitis (lifetime prevalence)**	1 (0.8)	35 (7.3)	1 (9.1)	0 (0.0)	17 (6.4)	0 (0.0)
**Obesity (BMI ≥30)**	0 (0.0)	10 (2.1)	0 (0.0)	6 (2.1)	12 (4.6)	0 (0.0)
**Current depression**	11 (8.9)	36 (7.5)	0 (0.0)	7 (2.4)	31 (11.7)	1 (14.3)
**Mental well-being**	4 (3.2)	26 (5.4)	0 (0.0)	10 (3.5)	22 (8.3)	0 (0.0)
**Social support**	0 (0.0)	9 (1.9)	0 (0.0)	0 (0.0)	9 (3.4)	1 (14.4)
**Current smoking**	0 (0.0)	13 (2.7)	0 (0.0)	0 (0.0)	11 (4.2)	0 (0.0)
**At-risk alcohol consumption**	2 (1.6)	51 (10.6)	0 (0.0)	6 (2.1)	33 (12.5)	0 (0.0)
**Sport participation**	0 (0.0)	13 (2.7)	0 (0.0)	0 (0.0)	3 (1.1)	0 (0.0)
**Influenza vaccination (2011/2012)**	0 (0.0)	20 (4.2)	0 (0.0)	1 (0.3)	9 (3.4)	0 (0.0)

SAQ-Web = self-administered web survey; SAQ-Paper = self-administered paper mail questionnaire; CATI = computer-assisted telephone interview.
