# Supplementary figures and images for: Mixing modes in a population-based interview survey: comparison of a sequential and a concurrent mixed-mode design for public health research
Source: Arch Public Health. 2018 Jan 4;76:8. doi: 10.1186/s13690-017-0237-1 (PMC5791202; doi:10.1186/s13690-017-0237-1)

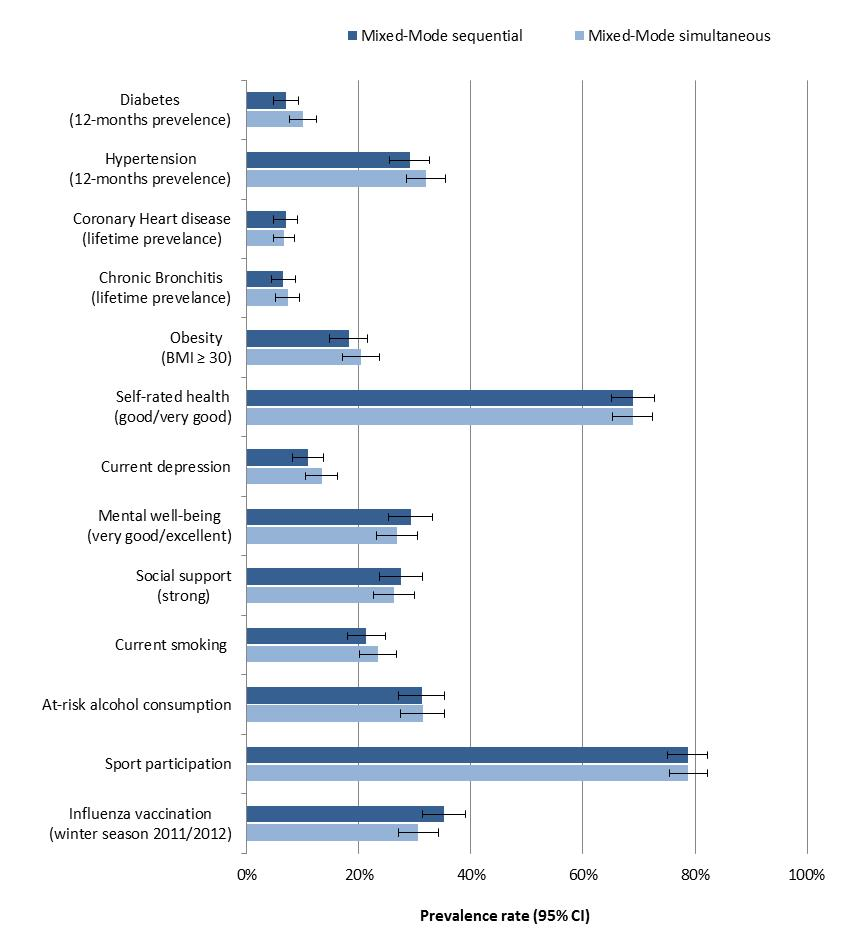

Supplement: Supplementary file 2 — Model-adjusted prevalence rates (95% confidence intervals) for basic health indicators by mixedmode design, adjusted for age, sex, marital status, household type, education, income, employment status, and migration background. (TIFF 208 kb) [file 13690_2017_237_MOESM2_ESM.tif]
